# Seasonal Variations in the Diet and Foraging Behaviour of Dunlins *Calidris alpina* in a South European Estuary: Improved Feeding Conditions for Northward Migrants

**DOI:** 10.1371/journal.pone.0081174

**Published:** 2013-12-03

**Authors:** Ricardo C. Martins, Teresa Catry, Carlos D. Santos, Jorge M. Palmeirim, José P. Granadeiro

**Affiliations:** 1 Centro de Biologia Ambiental/Museu Nacional de História Natural e da Ciência, Universidade de Lisboa, Lisboa, Portugal; 2 Centro de Estudos do Ambiente e do Mar/Museu Nacional de História Natural e da Ciência, Universidade de Lisboa, Lisboa, Portugal; 3 Department of Migration and Immuno-ecology, Max Planck Institute for Ornithology, Radolfzell, Germany; 4 Centro de Biologia Ambiental, Departamento de Biologia Animal, Faculdade de Ciências, Universidade de Lisboa, Lisboa, Portugal; 5 Centro de Estudos do Ambiente e do Mar, Departamento de Biologia, Universidade de Aveiro, Aveiro, Portugal; Pennsylvania State University, United States of America

## Abstract

During the annual cycle, migratory waders may face strikingly different feeding conditions as they move between breeding areas and wintering grounds. Thus, it is of crucial importance that they rapidly adjust their behaviour and diet to benefit from peaks of prey abundance, in particular during migration, when they need to accumulate energy at a fast pace. In this study, we compared foraging behaviour and diet of wintering and northward migrating dunlins in the Tagus estuary, Portugal, by video-recording foraging birds and analysing their droppings. We also estimated energy intake rates and analysed variations in prey availability, including those that were active at the sediment surface. Wintering and northward migrating dunlins showed clearly different foraging behaviour and diet. In winter, birds predominantly adopted a tactile foraging technique (probing), mainly used to search for small buried bivalves, with some visual surface pecking to collect gastropods and crop bivalve siphons. Contrastingly, in spring dunlins generally used a visual foraging strategy, mostly to consume worms, but also bivalve siphons and shrimps. From winter to spring, we found a marked increase both in the biomass of invertebrate prey in the sediment and in the surface activity of worms and siphons. The combination of these two factors, together with the availability of shrimps in spring, most likely explains the changes in the diet and foraging behaviour of dunlins. Northward migrating birds took advantage from the improved feeding conditions in spring, achieving 65% higher energy intake rates as compared with wintering birds. Building on these results and on known daily activity budgets for this species, our results suggest that Tagus estuary provides high-quality feeding conditions for birds during their stopovers, enabling high fattening rates. These findings show that this large wetland plays a key role as a stopover site for migratory waders within the East Atlantic Flyway.

## Introduction

Each year, millions of waders undertake long distance migratory movements between high-latitude breeding areas and temperate or tropical wintering grounds [1]. During these periods, most species depend on a network of wetlands along their flyways [1] where they stopover to rebuild their reserves and roost [2].

As most migratory taxa, waders have to cope with the challenge of finding food in a variety of habitats, experiencing dramatically different environmental conditions. Feeding resources, in particular, show high variability in composition and availability across the latitudinal gradient of breeding, stopover and wintering areas used by waders [3].

In estuarine and coastal wetlands, prey availability also varies seasonally, with prey biomass usually peaking during the warmer periods of the year [4]. In order to adapt to these rather short-termed variations in prey abundance and accessibility, waders generally show a strong behavioural plasticity, allowing them to explore a wide range of feeding resources during their annual cycle (e.g. [5], [6]).

The Dunlin (*Calidris alpina*) is a migratory wader, with circumpolar breeding range and a wide wintering distribution along temperate and subtropical coastlines of the Northern hemisphere [1]. As other calidrid species, dunlins possess very sensitive bills adapted for tactile foraging [7], mainly on polychaetes, molluscs and crustaceans [8]. However, they have been traditionally classified as mixed foragers, being able to alternate between visual and tactile foraging modes in response to variable environmental conditions. Indeed, previous studies on dunlins documented shifts in their diet and/or foraging strategy in response to factors such as sediment penetrability [9]–[11], light conditions [12]–[14] and prey availability [15]–[21]. Dunlins are then expected to take advantage of seasonal peaks of food abundance, in particular if such changes occur during periods of higher energetic demand, such as during migratory periods. Some authors have reported seasonal variations in the diet of dunlins [21]–[24], but the proximate factors of such variations are still poorly understood. Furthermore, no studies have focused on the role that changes in prey availability, in particular at the sediment surface, can have both in foraging strategies and diet and in the energy intake rates obtained by dunlins with different energetic constrains, such as wintering and passage migrant birds using the same intertidal flats.

The Tagus estuary, Portugal, holds internationally important numbers of wintering dunlins (more than 1% of the population [1]), with an overwintering population of ca. 10,000 individuals [25]. It is also a relevant stopover area for dunlins migrating along the East Atlantic Flyway, harboring an average of about 15,000 birds in the peak of northward migration [25]. Dunlins wintering in Tagus estuary include birds that breed in Scandinavia (*C. a. alpina*) and in the Baltic region and in the UK (both *C. a. schinzii*) [1], [26]. Northward migrants are thought to originate mostly from Mauritania, using the Tagus as stopover area when travelling to breeding grounds in Iceland (*C. a. schinzii*) [1], [27]. Wintering dunlins are in the estuary mainly from November to early-April, while most northward migrants arrive in large numbers in the second half of April [27].

Despite its importance as a stopover area, the great majority of the studies in the Tagus estuary have focused in wintering populations (e.g. [11], [12], [28]), so there is little information about wader foraging behaviour and feeding conditions during migratory periods [23].

In this study we aimed to (1) examine the differences in foraging behaviour and diet of wintering and northward migrating dunlins in the Tagus estuary and (2) investigate the extent to which these are influenced by seasonal variations in invertebrate availability. Additionally, we (3) calculated the energy intake rates achieved by dunlins in each season in light of their expected energetic demands and (4) discuss the significance of the improved feeding conditions provided by this temperate estuary for northward migrants in the context of the East Atlantic Flyway.

## Methods

### Ethics statement

Although our study area is included in a Special Protected Area for birds (*Zona de Protecção Especial do Estuário do Tejo*), the fieldwork carried out in this study did not require any legal permission from the Nature Reserve authorities, as it did not involve capture or manipulation of birds. Furthermore, the observation of birds, at a distance, did not cause any disturbance and Dunlin is not an endangered species. It is considered a “species of Community interest” according to the Portuguese law (DL 49/2005, that transposes the European Birds' Directive) and has the conservation status of “Least Concern” according to both the Red Book of the Vertebrates of Portugal [29] and the IUCN Red List of Threatened Species [30].

### Study area

**Figure 1:**
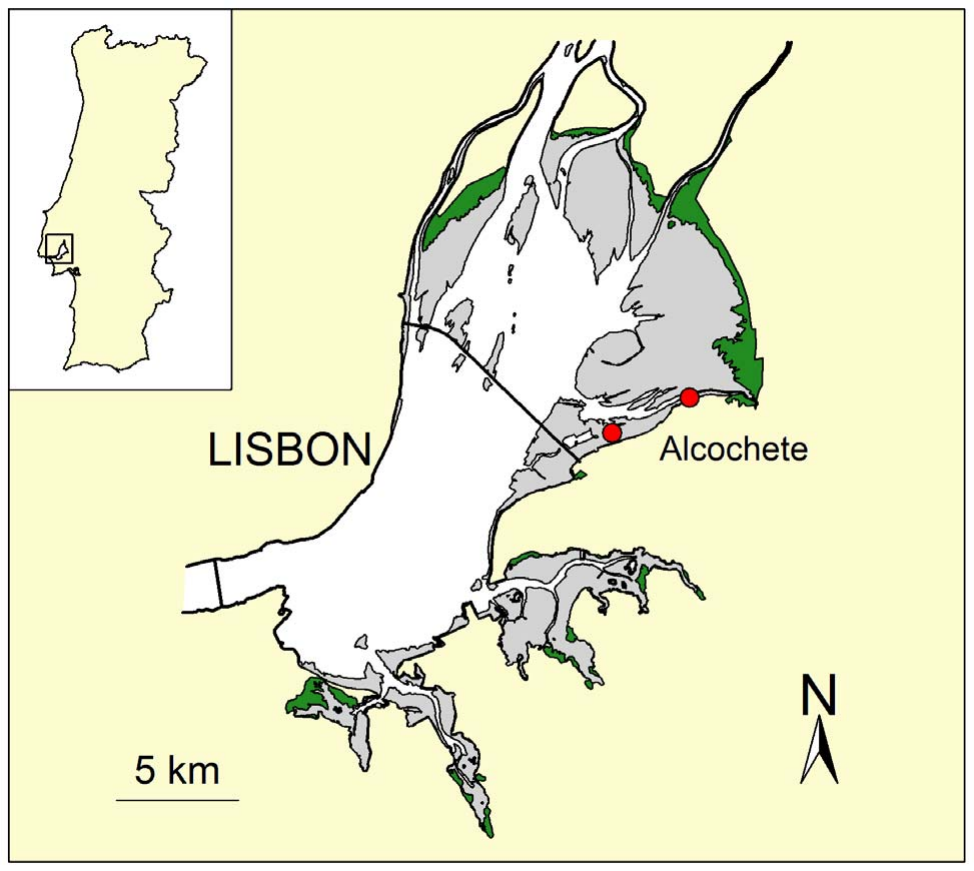
Map of the Tagus estuary, showing the location of the study area. Red dots represent the selected sectors of intertidal mudflat. Grey shading represent the intertidal area and green areas represent saltmashes.

This study was carried out in the Tagus estuary, Portugal (38°45′N, 09°00′W; Figure 1), which covers a total area of ca. 13,000 ha, largely dominated by mudflats, but also including oyster beds and sandy flats [31].

Our study area was located in the southern margin of the estuary (Figure 1), where two sectors of intertidal mudflat were selected near the shoreline (ca. 200 m). Western and eastern sectors had an exposure period of approximately three and five hours in spring tides (amplitude >2.7 m) and cover 4.5 and 18 ha, respectively. The sediment consisted of mud (>95% of particles <63 µm [32]) and the mean low tide densities of dunlins recorded in the study area during winter and spring were of 5.6±1.2 (SE) and 3.4±2.5 (SE) birds/ha (n = 8 and 6 counts), respectively. These sectors are representative of the intertidal areas commonly used by dunlins in the Tagus estuary [31]. All field work procedures were replicated in both sectors.

### Bird observations

Haphazardly chosen dunlins were video-taped during diurnal low tides (±2 h from low tide peak) in winter (Jan-Feb of 2009 and 2010) and spring (mid-Apr-May of 2009 and 2010) while foraging in the study area. Recordings were carried out with a digital camcorder (NV-DS15, Panasonic) with a 20× optical zoom, equipped with a 1.4× adapter. Birds were filmed at close distances (from 8 to 30 m, mean ± SD  = 17.7±5.5 m, n = 155). In general, birds were numerous and moved in the same direction along the shoreline. Therefore, it is highly unlikely that pseudoreplication (i.e. filming the same individual more than once) affected our dataset and conclusions to any significant extent. A total of 99 and 56 one-minute good-quality videos were obtained, respectively in winter and spring (recordings of poor quality were discarded). Sampling periods were chosen to ensure that birds recorded in winter belong to the local wintering population and those sampled in spring belong mainly to the African-wintering populations that stopover in Tagus estuary during their northward migration.

### Foraging behaviour

Video recordings of foraging dunlins were examined in slow-motion to quantify the number of steps and number of pecks per minute. Three different types of pecks were considered (see Videos S1 and S2): (1) superficial pecks – when only the tip of the bill (<5 mm) is inserted in the sediment; (2) probes – when more than 5 mm of the bill is inserted in the sediment; and (3) sweeps – essentially used as a visual technique, comprising a very fast sequence of opening and closing movements of the flexible tip of the bill (>5 cycles/sec) while making small range (<3 cm) radial movements of the neck. Sweeps were usually performed in the water film for capturing shrimps (Video S2, sec 21–38). No evidence of biofilm grazing was observed in the videos [33].

### Characterization and quantification of diet

Prey identity and consumption rates were assessed from video recordings and complemented with dropping analyses. Analysis of video recordings allowed the accurate distinction between filiform (worms and siphons of bivalves) and non-filiform prey items (crustaceans, bivalves and gastropods), as filiform prey are usually pulled out of the sediment and are visible even when extracted by deep probing.

The specific identification of filiform prey was possible in a subset of recordings (ca. 20% in each season) obtained at a short distance, and revealed that birds were consuming the polychaete *Hediste diversicolor* and siphons of the bivalve *Scrobicularia plana*, in both seasons. Therefore, prey-specific consumption rates of filiform prey were calculated for all video recordings assuming the relative occurrence of *S. plana* siphons or *H. diversicolor* in this subset of recordings, in each season.

Among non-filiform prey, isopodes can also be identified in video recordings [17], as well as shrimps, whose capture by dunlins involves a specific and easily recognizable behaviour (Video S2, sec 21–38; see also [21]). However, the consumption of entire bivalves and gastropods could not be well assessed even with the best quality recordings. Although consumption of whole bivalves by dunlins results in the typical swallowing movements, these prey are difficult to be confirmed in recordings (but see two exceptions on Video S1) because they are often ingested during continuous probing. Gastropods consumed by dunlins in the study area (*Hydrobia ulvae*) live on the sediment surface but due to their small size they are quickly collected with superficial pecks and ingested without the typical swallowing movement (see also [17]) and consequently can not be detected in video recordings. Furthermore, dunlins also use superficial pecks to capture (e.g.) retractable prey available at sediment surface (worms and bivalve siphons) and we were often unable to distinguish unsuccessful attempts to capture such retractable prey from captures of *H. ulvae*.

Due to these difficulties, the quantification of the consumption of bivalves and gastropods was supported by the analysis of 80 and 148 dunlin droppings, collected in winter (Jan-Feb 2010, in eight sessions) and spring (mid-Apr-May 2010, in 14 sessions) periods, respectively. Droppings were collected in the study area immediately after video recording foraging birds, and at least 30 minutes after a flock arrived in the area. Dunlin droppings could be easily identified in the sediment by their size and shape, and there were no other similar-sized species in the study area that could cause identification issues. Whole droppings were carefully collected from the sediment surface, stored in individual containers and frozen at -18°C prior to analysis. In the laboratory, droppings were examined with a stereomicroscope, and prey species were identified using diagnostic remains.

We assumed that all ingested items classified in video recordings as unidentified non-filiform prey were entire *S. plana*. This is supported mainly by the fact that the capture of these items resulted exclusively from probing and that droppings did not contain remains of any other non-filiform buried-living prey. Accordingly, the seasonal differences in the occurrence of shell fragments of *S. plana* in droppings (73.8% in winter and 19.6% in spring) were similar to those found in the consumption rate of those unidentified non-filiform items in recordings (see results).

The quantification of *H. ulvae* in the diet of dunlins was based only in dropping analyses and is expressed as a frequency of occurrence.

### Prey sampling and availability

The density of benthic prey was determined in the study area by randomly taking 100 and 114 sediment cores (86.6 cm^2^, 30 cm deep) in late winter (early March 2010) and spring (end of May 2010), respectively. The upper 5 cm of each core was sieved through a 0.5 mm mesh (to ensure that small prey in the top layer could be quantified), whereas a 1 mm mesh was used to sieve the remaining sediment. Shrimps available at the surface water film were unlikely to be adequately sampled with sediment cores. Therefore we used a wood enclosure (75×75 cm wide and 20 cm high) that was randomly thrown to the surface of the sediment, 40 and 47 times during the winter and spring periods, respectively. The whole area of the enclosure was then carefully sieved with a square-shaped landing net with a mesh of 1 mm, collecting all shrimps in the sediment surface and water film. Invertebrates collected with both techniques were stored in 70% alcohol and later identified using stereomicroscope, and their densities were calculated.

Prey biomass (expressed in AFDW/m^2^) was estimated by measuring their body/structure size and then using published relationships between size and biomass for each species (Table 1). Some *H. diversicolor* were not intact and therefore we estimated the total body length using published relationship between mandible and body lengths (Table 1). The length of *S. plana* siphons was estimated from regressions with shell size, following Zwarts et al. [34] (Table 1).

**Table 1:** Equations used to calculate biomass (ash free dry weight, AFDW) of Dunlin invertebrate prey.

	Equation	Source
*Hediste diversicolor*	TL = 40.173ML−3.4225	[64]
	AFDW = 10^(2.53LOG(TL)−5.94)^×0.771×1000	[23]
*Scrobicularia plana*	AFDW = 10^(2.49LOG(APL)−4.57)^×0.795×1000	[23]
Siphons of *S. plana*	SLS = 0.9APL+1.4	[34]
	AFDW = SLS×0.00014APL^1.69^	[34]
*Hydrobia ulvae*	AFDW = 0.0154SL^2.61^	[11]
*Crangon crangon*	AFDW = 0.2((TL+1.1295)/4.7906)^3.0725^	[46]

TL- total length (mm); ML- mandible length (mm); AFDW- biomass, expressed as ash free dry weight (mg); APL- antero-posterior length of the shell (mm); SLS- siphon length at surface (mm); SL- shell length.

There is no detailed information on seasonal variations in size-biomass relationships for all dunlin prey in the Tagus estuary. However, Guerreiro [35], in a study with *S. plana* in Portuguese estuaries, showed minor winter to spring variations in such relationship in comparison of those obtained in higher latitudes [36]. Furthermore, Guerreiro [35] also showed that the smaller sizes of *S. plana* (corresponding to the sizes consumed by dunlins) are virtually not affected by seasonal variations in size-biomass relationships. Therefore, assuming that this also applies to other prey, it is unlikely that our estimates of invertebrate biomass are significantly affected by such effect.

We considered that all invertebrates present in the upper 5 cm of core samples (plus shrimps) were available for dunlins, with the exception of bivalves and polychaetes larger than 13 and 66 mm, respectively, as they are not consumed by dunlins [11]. We also considered that all polychaetes less than 66 mm long and the siphons of bivalves present in the lower portion of the core were available, as their surface activity [37], [38] makes them accessible to dunlins [11], [23].

The availability of *H. diversicolor* and siphons of *S. plana* at the surface of the sediment (nr. of active items/m^2^) was further quantified with video recordings, in winter and spring. A digital camcorder (NV-DS15, Panasonic) was used to record invertebrate activity in 50×50 cm areas of exposed sediment, during 6 min, within 2 h from the low tide. A total of 17 and 56 recordings were obtained in winter (Jan-Feb 2010) and spring (Apr-May 2010), respectively.

Each video recording was analysed in a computer and the number of *S. plana* siphons and *H. diversicolor* visible at the surface in each sampling square were counted at intervals of 0.5 min. In all videos, we excluded the first 2 min and the last 1.5 min of the recordings, to avoid any observer effect. We recorded the highest count of each prey item in each video recording, and then averaged these values across all videos, as an estimate of surface prey availability. The biomass of *S. plana* siphons and *H. diversicolor* available at the surface was estimated by multiplying the average number of visible items (obtained from video recordings, in items/m^2^), by the season-specific average biomass of each prey, obtained from core sampling.

### Energy intake rates of dunlins

Energy intake rates of dunlins were obtained by multiplying the number of items consumed per minute (estimated from video recordings) by their specific average energetic content. The ingestion of individual *H. ulvae* could not be detected in video recordings (see above) and therefore we estimated their consumption rate in each season by multiplying the rate of superficial pecks identified in video recordings as potential captures of *H. ulvae* by the frequency of occurrence of this prey in droppings.

The average energy content of each prey was calculated using published information relating structural size with biomass (Table 1) and then considering that one gram of ash free dry weight (AFDW) of each prey corresponds to about 23 Kj [39].

Average sizes of consumed *S. plana* and *H. ulvae* were obtained from Santos et al. [11] (Table 2) as we confirmed that the size distribution of these two prey species in our study is very similar of that obtained by those authors, within the size classes consumed by dunlins [11]. The size of *H. diversicolor*, the shrimp *Crangon crangon* and siphons of *S. plana* consumed by dunlins was estimated in relation to culmen length, from our best-quality videos, when these items were held by the tip of the bill immediately before ingestion (Table 2). Average culmen length of dunlins in the Tagus estuary is 32.3 mm (SD  = 2.9) and 30.8 mm (SD  = 2.8) for wintering and northward migrating birds, respectively (authors' unpublished data).

**Table 2:** Mean sizes (± SD, sample sizes in parenthesis) and mean individual energy content of prey consumed by dunlins in winter and spring.

	Size (mm)		Energy content (J)	
	Winter	Spring	Winter	Spring
*H. diversicolor*	15.4±4.7 (4)	24.6±9.9 (18)	20.7	67.4
*C. crangon*	—	13.5±2.4 (11)	—	142.2
Siphons of *S. plana*	21.1±10.8 (19)	44.1±10.5 (5)	28.2	46.8
*S. plana*	7.1±0.4 (169)	—	64.8	—
*H. ulvae*	1.8±1.5 (216)	—	1.64	—

Consumed sizes of *S. plana* and *H. ulvae* were obtained from Santos et al. [11]. Size of *S. plana* refers to antero-posterior length of the shell. Size of siphons refers to siphon length at surface. Size of *H. ulvae* refers to shell length.

### Statistical analyses

Most of the data failed to meet the assumption of normality and homocedasticity (even after transformation) and therefore we used Mann-Whitney tests to compare diet and behavioural parameters, as well as prey availability in winter and spring periods.

## Results

### Foraging behaviour and diet of dunlins

**Figure 2:**
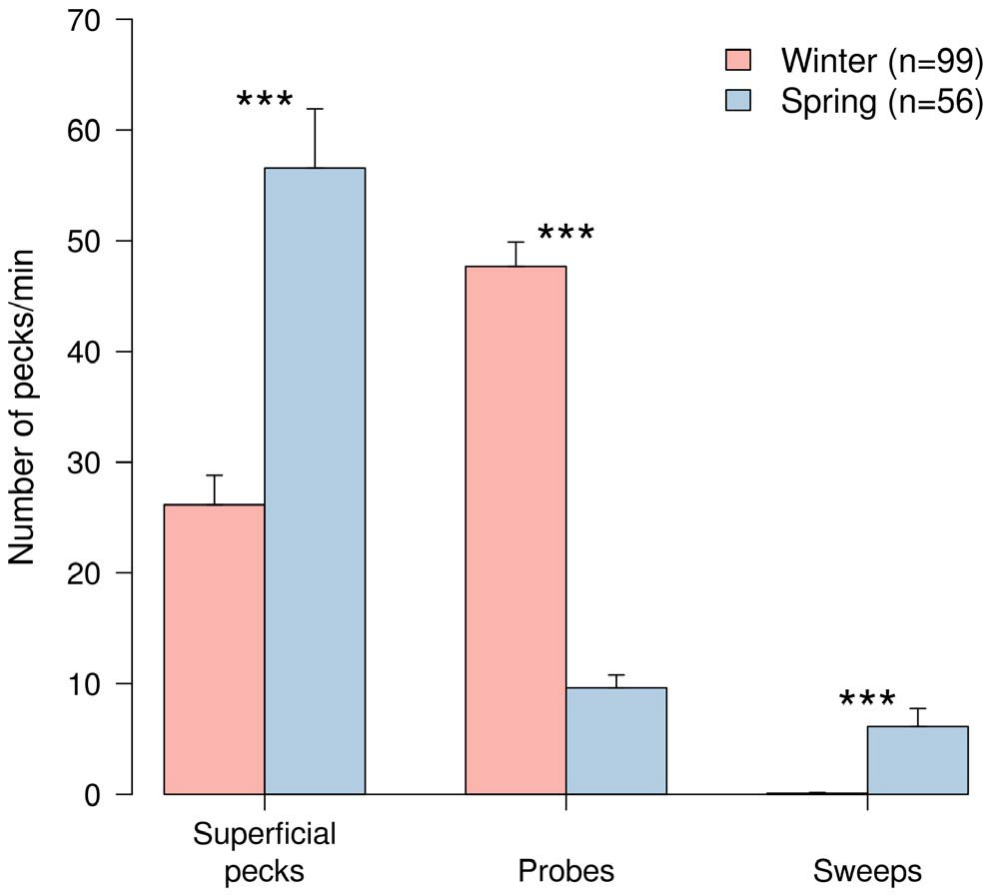
Mean rate of superficial pecks, probes and sweeps of foraging dunlins in winter and spring. Values represent mean ± SE. Differences between seasons were tested with Mann-Whitney test (*** p<0.001).

Foraging dunlins showed a significantly lower step rate in winter than in spring (134±6.0 (SE) steps/min in winter and 163±6.1 (SE) steps/min in spring; Mann-Whitney test: U = 1952.5, p<0.01). The total number of pecks (superficial pecks, probes and sweeps) did not vary between seasons (74.0±2.8 (SE) pecks/min in winter and 72.3±5.5 (SE) in spring; Mann-Whitney test: U = 3089.5, p = 0.238). However, we found significant differences in the type of feeding techniques used from winter to spring (Figure 2). In fact, superficial pecking rate increased significantly in spring, whereas the opposite trend was observed for probing (Figure 2). Sweeping was only observed in spring. These differences were also significant when each sampling year was analysed separately (Mann-Whitney tests, all p<0.001).

**Figure 3:**
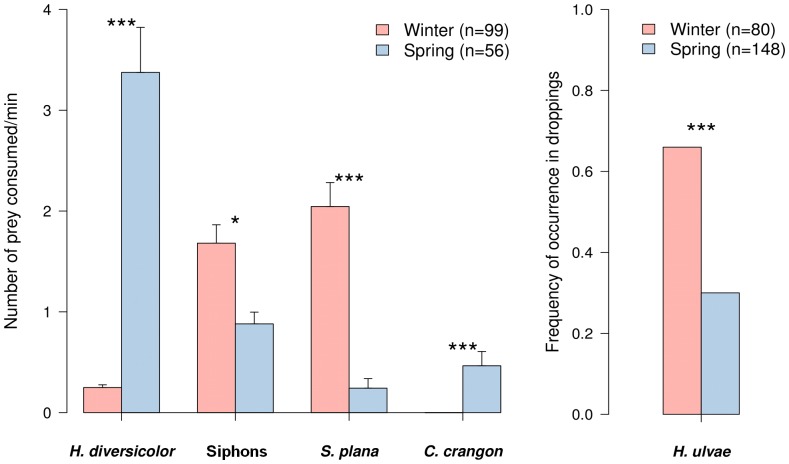
Diet of Dunlin in winter and spring assessed by (A) field observations and (B) dropping analyses. A: Consumption rate (prey/min) of main prey. B: Occurrence of shell remains of *H. ulvae* in Dunlin droppings. Values represent mean ± SE. Differences between seasons were tested with (A) Mann-Whitney (B) and Chi-squared tests (* p<0.05; *** p<0.001).

The diet of dunlins also differed significantly between seasons (Figure 3). In winter, dunlins consumed mainly *H. ulvae* and *S. plana* (small entire individuals and siphons), whereas in spring the main prey was *H. diversicolor*, and to a lesser extent *S. plana* siphons. The shrimp *C. crangon* was consumed only in spring and at a relatively low rate (Figure 3A). Videos S1 and S2 show examples of dunlins catching different prey.

### Prey availability

*H. ulvae* was the most abundant prey species in the study area, but the harvestable biomass for dunlins was dominated by *H. diversicolor*, especially in spring (Table 3). *S. plana*, siphons of *S. plana*, and *H. ulvae* had similar contributions to the available biomass and *C. crangon* was virtually irrelevant (Table 3).

**Table 3:** Seasonal variation in harvestable density and biomass of main Dunlin prey species in winter and spring.

	Density (indiv./m^2^)			Biomass (g of AFDW/m^2^)		
	Winter	Spring	M-W test	Winter	Spring	M-W test
*H. ulvae*	1947±153	3768±263	U = 3362 **p<0.001**	0.25±0.02 (15%)	0.27±0.02 (6%)	U = 5300 p = 0.377
*S. plana* ^1^	367±51	304±41	U = 5787 p = 0.845	0.20±0.03 (12%)	0.49±0.07 (11%)	U = 3945 **p<0.001**
Siphons of *S. plana* ^2^	89.5±11.6	181.6±11.7	U = 3187.5 **p<0.001**	0.26±0.03 (16%)	0.43±0.03 (10%)	U = 4070 **p<0.001**
*H. diversicolor* ^3^	310±32	381±31	U = 4868 p = 0.06	0.94±0.10 (57%)	3.34±0.27 (73%)	U = 2734 **p<0.001**
*C. crangon*	0.04±0.04	11.5±5.4	U = 5.5 **p<0.001**	<0.01±<0.01 (0%)	0.05±0.01 (1%)	U = 5 **p<0.001**
Total Biomass				**1.65**	**4.58**	

Means are represented ± SE. Relative contribution of each prey for the total biomass in each season is shown between brackets. Differences between seasons were tested with Mann-Whitney test (M-W test). Data were obtained from 100 core samples in winter and 114 in spring, except for *Crangon crangon*, which resulted from 40 and 47 sampling squares, respectively (see methods for further details). ^1^ Juvenile individuals, considered to be reachable and ingestible (whole) by dunlins, as they lay in the upper sediment fraction (0–5 cm deep) and are small (<13 mm). ^2^ Siphons of the individuals that are out of reach of a Dunlin's bill, as they lay in the lower sediment fraction (5–30 cm deep), corresponding mostly to large (>30 mm) and adult individuals. ^3^ Individuals longer than 66 mm were excluded.

There was a noticeable increase (>2.5×) in the overall harvestable biomass from winter to spring (Table 3). This was mostly due to increases in the size of *H. diversicolor* and (juvenile) *S. plana* and in the number of adult *S. plana* (which are inaccessible to dunlins but provide larger siphons at the surface).

The availability of *H. diversicolor* and *S. plana* siphons at the sediment surface also increased significantly from winter to spring (Table 4). Illustrative examples of this result are shown on Videos S3 and S4. Although such increases were similar in terms of numerical importance between these two prey items (12.5× in *S. plana* siphons and 15.5× in *H. diversicolor*), the corresponding biomass values were of rather different magnitude: 10× in *S. plana* siphons and 44× in *H. diversicolor*.

**Table 4:** Seasonal variations in the surface availability of *Hediste diversicolor* and siphons of *Scrobicularia plana* and their corresponding biomass.

	Surface availability (indiv./m^2^)			Biomass (mg of AFDW/m^2^)	
	Winter (n = 17)	Spring (n = 56)	M-W test	Winter	Spring
*H. diversicolor*	0.4±0.4	6.4±2.2	U = 354 **p<0.05**	1.2±1.2	53.4±19.2
Siphons of *S. plana*	1.1±0.7	13.7±3.1	U = 201 **p<0.001**	3.2±1.9	32.3±7.2

Means are represented ± SE. Differences between seasons were tested with Mann-Whitney test (M-W test). Biomass was estimated by multiplying the surface availability by the mean individual biomass of each prey in each season (obtained from core sampling).

### Energy Intake Rates of dunlins

**Figure 4:**
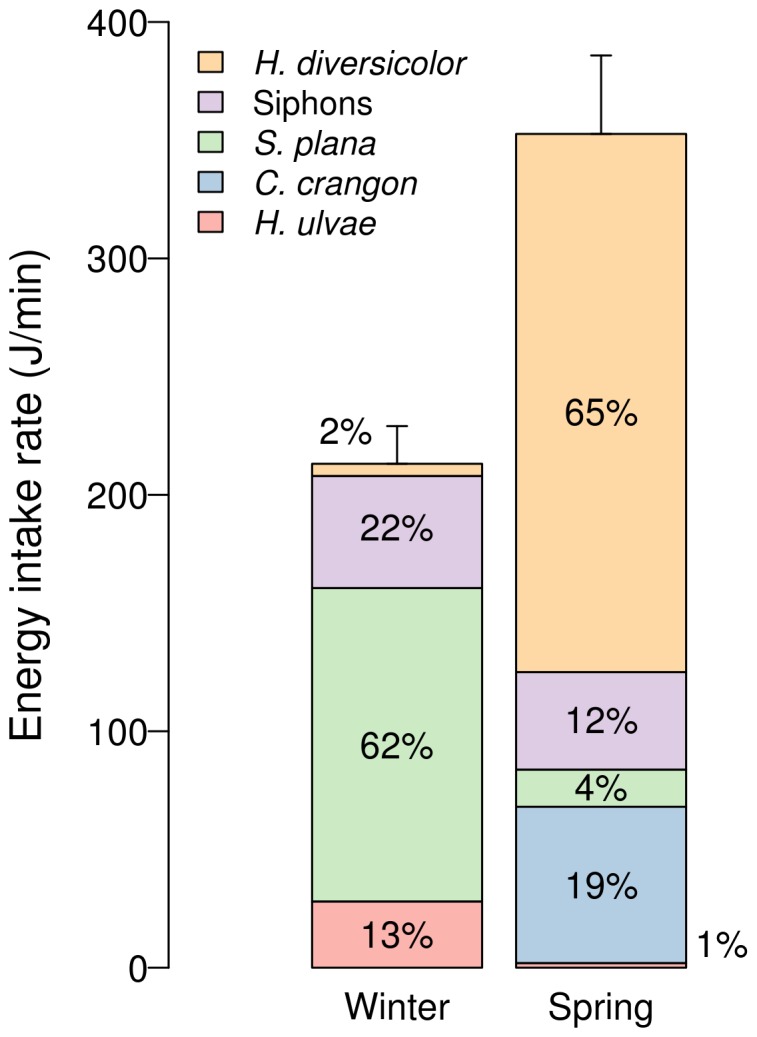
Mean energy intake rates achieved by foraging dunlins in winter and spring (J/min). Relative contribution of different prey is shown, as percentage. Error bars represent the SE for pooled prey.

In spring dunlins achieved energy intake rates 65% higher than wintering birds (Figure 4; Mann-Whitney test: U = 1877, p<0.001). In winter, whole *S. plana* was clearly the main source of energy whereas *H. diversicolor* was the least important. In contrast, in spring *H. diversicolor* contributed the most energy while whole *S. plana* played a relatively minor role (Figure 4).

## Discussion

### Foraging behaviour and diet of wintering and northward migrating dunlins

In this study we found clear differences between the foraging behaviours and diets of the Dunlin populations wintering in the Tagus estuary and those using it in spring, during their northward migration. In winter, birds predominantly adopted a tactile foraging technique (probing), mainly used to search for juvenile *S. plana*. The lower step rate observed during this period results from the prolonged searching times associated with the probing technique [21]. Visual methods (superficial pecking) were also used in winter, to collect the gastropod *H. ulvae* and siphons of adult *S. plana*. In contrast, in spring dunlins generally used a visual foraging strategy (superficial pecking and sweeps), mostly to consume *H. diversicolor*, but also *S. plana* siphons and *C. crangon*. The higher step rate of foraging birds, observed in spring, is characteristic of visual techniques, as it increases the encounter rate and capture success of retractable items available at the surface [40].

Diet composition of dunlins recorded in this study is mostly in accordance with other studies in northern and southern European estuaries. In fact, the prey most frequently reported in the literature include polychaetes (mainly *H. diversicolor* but also *Nephtys sp.* and other *taxa*), bivalves (*S. plana*, *Cerastoderma edule* and *Macoma baltica*) and the widespread and abundant gastropod *H. ulvae* [11], [22]–[24], [41]–[44]. Seasonal shifts in dietary regimes have been previously described for dunlins, namely the higher predation upon *H. diversicolor* and crustaceans (like *C. crangon*) during migratory periods [20], [21]. In particular, the increase of *H. diversicolor* consumption between winter and spring, concurrently with a decrease in the intake of gastropods and bivalves, observed in our study, were previously reported by Worral [24] and Moreira [23].

### The influence of seasonal variation in food availability in the diet and foraging behaviour of dunlins

In this study we found a substantial increase in the harvestable prey biomass for waders from winter to spring, as described in previous studies carried out in temperate estuaries [36], [45]. This variation resulted from a combined effect of increased densities (siphons of *S. plana*) and larger size of individual prey (in juvenile *S. plana* and *H. diversicolor*).

We found a very marked seasonal increase in the surface availability of *S. plana* siphons and *H. diversicolor* (12.5× and 15.3×, respectively), mostly due to a much higher surface activity in spring, and to lesser extent to variation in densities, which increased quite modestly (only 2.0× and 1.2×, respectively). Our study revealed important seasonal variations in surface behaviour in these two species, which imply a higher exposition to predation by birds. The increase in activity probably resulted from a higher prevalence of deposit feeding mode in *S. plana* [38] and an intensification of foraging activity of *H. diversicolor*, due to higher temperatures [37].

These observations pooled together strongly suggest that seasonal variations in biomass and activity of invertebrate key prey played a critical role in shaping the diet and foraging behaviour of wintering and northward migrating dunlins; feeding opportunities were much greater for the latter. The combined effect of the seasonal increase in surface activity and individual size of *H. diversicolor* resulted in a very strong surge in their biomass available at the sediment surface, certainly changing their profitability in relation to other prey. The shrimp *C. crangon* also represented an important addition to the diet of northward migrating dunlins, contributing with 19% of the total energy intake, despite its low abundance and modest contribution to the overall available biomass (ca. 1%). The apparent preference for this prey seems to be mainly explained by their high individual energetic content [21], [46] (Table 2).

The spring increase in the availability of *H. diversicolor*, *S. plana* siphons and *C. crangon* at the sediment surface and its water film was likely responsible for the visual feeding behaviour observed in northward migrating dunlins, as these prey represented ca. 96% of both the consumed prey and energy intake rates in this season (Figure 4).

Our results support the idea that dunlins have a high foraging plasticity, efficiently exploiting a wide range of food resources while aiming at the most profitable prey. In this context, it should be noted that the dietary changes observed in our study do not seem to be a consequence of a decline in the availability of the prey preferred during the winter towards spring. Indeed, the preferred prey in winter (*H. ulvae, S. plana* and siphons) were mainly ignored by dunlins in spring, although their abundance remained stable or even increased. Dunlins are well adapted to tactile predation, holding high densities of sensory pits in the bill [7]. Nonetheless, evidence from studies comparing daytime and night-time foraging behaviour of dunlins suggests that visual predation tends to be the preferred foraging mode whenever conditions allow birds to use visual clues [12]–[14], as found in our study.

### Energy intakes and stopover importance of the Tagus estuary

In the Tagus estuary, northward migrating dunlins do not seem to be more constrained by feeding resources in relation to wintering birds. Due to higher prey availability at the sediment surface in spring, migrating birds achieved energy intake rates that were 65% higher than those wintering in the same estuary (353 J/min and 213 J/min, in spring and winter, respectively). But is this increment likely to meet the high energy demands of migrating birds [39] and is it enough to allow for a quickly refuelling?

Dunlins have an average body mass of 46 g and an estimated daily energy expenditure (DEE) in the temperate zone of ca. 139 Kj/day (assuming that DEE corresponds to 3× the basal metabolic rate [47]). This requires a gross intake of 163 Kj/day, assuming a digestive efficiency of 85% [47]. As dunlins in the Tagus estuary forage moving through the intertidal mudflats since the higher areas exposed until they cover again [48], they are able to feed for 7.5 h/tide. Therefore, wintering dunlins at the intake rate of 213 J/min (estimated in this study) obtain ca. 96 Kj in each diurnal tide, which corresponds to 59% of their daily requirements. Nocturnal energy intake rates are not known but dunlins are well adapted to tactile feeding [7] and usually forage at night [12], [13], [49]. Therefore, it is likely that they are able to obtain a significant part of their energy requirements during the night, as found in others waders wintering in the Tagus estuary [12]. To fulfil their daily energy requirements (assuming similar diurnal and nocturnal intakes rates), they would need to forage for 4.5 h of the 7.5 h of sediment exposure during a typical night time tidal cycle. Therefore, it seems that wintering dunlins can fulfil their energetic requirements in the intertidal area, and thus do not need to feed in supratidal areas during high-tide periods, such as the saltpans where they usually roost during these periods [50], [51].

The energy requirements of dunlins during northward migration are poorly known, as fattening rates vary according to their migration strategies and schedules [52], [53]. The maximum theoretic and empirical rates of weight gain calculated for most waders are around 4–5% of weight gain per day [53] and the highest rates obtained by dunlins in stopover areas are ca. 3.9% [54]. Fattening rates of this magnitude correspond to a weight gain of 1.8 g/day in dunlins, requiring an additional 82 Kj above maintenance levels, i.e. a total of 245 Kj/day (considering that each g of weight gain requires 45.7 Kj/day above maintenance costs [47]). Assuming the intake rate estimated for migrating dunlins in this study (353 J/min), birds can obtain about 159 Kj during diurnal tides. Therefore, to achieve the highest fattening rates during stopover, dunlins need an extra 86 Kj each night (245-159 Kj), or only 4.1 h/day of nocturnal feeding (out of 7.5 h). This value is less than that required by wintering birds to fulfil their energy requirements. These crude estimations suggest that the activity budgets of northward migrating dunlins are quite similar to those of wintering birds and therefore they seem to be able to achieve high fattening rates in the intertidal areas of the Tagus estuary, without the need to feed during high tide. In fact, shorebird fattening rates in temperate areas tend to be higher than in the tropics [52]–[56], due to the seasonal peaks in food availability, which are typical of temperate areas [3], [4], [36], [45], [57] and are coincident with migratory periods (this study, [58]).

Migratory wader populations are suffering a global decline [59] and the success of their migration relies on a network of high-quality stopover sites in-between breeding and wintering areas [60]–[62], where they can rapidly rebuild their condition and pursue their journey. The available evidence from regular wader counts suggests that the Tagus estuary plays a critical role as a stopover area for thousands of birds within the East Atlantic Flyway [1], [25], representing an important link between the wetland-rich northwestern Europe and the distant (albeit very large) wintering areas of the western coast of Africa, such as the Banc d'Arguin (Mauritania) and Guinea-Bissau. Our results indicate that Tagus estuary provides high-quality feeding areas for dunlins and probably other waders during northward migration. The long-term deterioration of feeding conditions for waders in stopover sites can cause changes in their migratory paths and timings [63] and consequently adversely affect the reproductive success of individuals [60], through carry-over effects. From a conservation stand-point it is crucial that large-scale impacting activities (such as extensive sediment dredging or reworking, infrastructure expansion into intertidal areas, roost/habitat degradation or over-shellfishing) are carefully controlled, to ensure that the quality of feeding conditions for migratory waders is not affected in this internationally important wetland.

## Supporting Information

Video S1 **Samples of six video recordings of dunlins foraging in the study area in winter.** The first four samples (sec 0–24) show dunlins probing to capture juveniles of the bivalve *Scrobicularia plana* (visible in sec 4 and 10), alternating with isolated superficial pecks (e.g. sec 7, 18 and 21), probably to collect the gastropod *Hydrobia ulvae*. The last two video samples (sec 24–34) show dunlins preying on siphons of adult *S. plana*. The use of slow motion is recommended.
(WMV)

Video S2 **Samples of six video recordings of dunlins foraging in the study area in spring.** The first four samples (sec 0–20) show dunlins preying on the polychaete *Hediste diversicolor* and the last two (sec 21–38) show the typical behaviour of dunlins preying on shrimps (*Crangon crangon*), which frequently jump out of the water, trying to escape (sec 24 and 33). The use of slow motion is recommended.
(WMV)

Video S3 **Samples (5 sec each) of five video recordings of the sediment (50×50 cm area) obtained in winter to record the surface availability of** ***Hediste diversicolor*** **and siphons of** ***S. plana*** **for dunlins.** Videos are shown in double speed to facilitate the detection of invertebrate activity. A single siphon is active in each of the two first samples.
(WMV)

Video S4 **Samples (5 sec each) of five video recordings of the sediment (50×50 cm area) obtained in spring to record the surface availability of** ***Hediste diversicolor*** **and siphons of** ***S. plana*** **for dunlins.** Videos are shown in double speed to facilitate the detection of invertebrate activity. Several items are visible in all samples. In first (sec 0–5) and third (sec 10–15) samples are present mainly siphons (whitish color), the remaining samples showing mainly small *H. diversicolor* (reddish-brownish color).
(WMV)
